# Meta-analysis and cost-effectiveness of ductoscopy, duct excision surgery and MRI for the diagnosis and treatment of patients with pathological nipple discharge

**DOI:** 10.1007/s10549-021-06094-x

**Published:** 2021-01-21

**Authors:** M. D. Filipe, S. I. S. Patuleia, M. R. Vriens, P. J. van Diest, A. J. Witkamp

**Affiliations:** 1 Department of Surgical Oncology, Cancer Centre, University Medical Centre, PO Box 85500, 3508 GA Utrecht, The Netherlands; 2 Department of Pathology, University Medical Centre, Utrecht, The Netherlands

## Abstract

**Introduction:**

Pathological nipple discharge (PND) is a common breast-related complaint for referral to a surgical breast clinic because of its association with breast cancer. The aim of this meta-analysis was to compare the diagnostic efficacy of magnetic resonance imaging (MRI) and ductoscopy in patients with PND. Additionally, we determined the most cost-efficient strategy for the treatment of PND and the detection of breast cancer in PND patient without radiological suspicion for malignancy.

**Materials and methods:**

PubMed and EMBASE were searched to collect the relevant literature from the inception of both diagnostic methods until January 27th 2020. The search yielded 815 original citations, of which 10 studies with 894 patients were finally included for analysis. Costs of ductoscopy, MRI and duct excision surgery were obtained from the UMC Utrecht as established in the year 2019. These costs included: medical personnel, overhead costs, material costs and sterilisation costs.

**Results:**

The meta-analysis showed no significant difference in sensitivity between ductoscopy (44%) and MRI (76%) for the detection of malignancy in patients with PND. However, ductoscopy (98%) had a statistically significantly higher specificity than MRI (84%). Individual costs were €1401.33, €822.13 and €6494.27 for ductoscopy, MRI and duct excision surgery, respectively. Full diagnostic strategy involving ductoscopy was on average €1670.97, while with MRI it was €2070.27.

**Conclusion:**

Patients undergoing MRI are more often (false) positive which more often leads to duct excision surgery referrals compared to ductoscopy. This makes ductoscopy significantly more cost-effective compared MRI in patients with PND without radiological suspicion for malignancy.

**Supplementary Information:**

The online version of this article (doi:10.1007/s10549-021-06094-x) contains supplementary material, which is available to authorized users.

## Introduction

Pathological nipple discharge (PND) is defined as unilateral, spontaneous and bloody or serous discharge, usually arising from a single duct orifice of the nipple. After pain and palpable lumps, PND is the third most common breast-related complaint [1] and it accounts for 3–5% of surgical breast clinic referrals [2–5] Even though it is considered a red-flag symptom for breast cancer, the most common causes of PND are benign, namely ductal ectasia and intraductal papillomas [6, 7].

Traditionally, patients suffering PND are offered major duct excision surgery to rule out malignancy [6, 8, 9], which occurs in only 5–8% [4, 10, 11]. This means that around 92–95% of these operations are performed for non-malignant causes. However, although invasive, the advantage of major duct excision is that it can also be helpful to treat PND itself.

Magnetic resonance imaging (MRI) has more recently shown to be a sensitive tool for the detection of malignancy in patients with PND. However, MRI has some shortcomings, namely in the detection of small lesions and in differentiating benign from malignant masses [9, 12]. Therefore, the value of MRI is limited in patients with PND and core needle biopsy or surgical excision is still necessary when MRI shows a suspicious lesion [13, 14]. This not only leads to a longer diagnostic path but is also accompanied by accumulation of costs.

Ductoscopy is a minimally invasive micro-endoscopic technique providing real-time visualization of the milk ducts of the breast. This procedure is performed under local anaesthesia at the outpatient clinic and is currently used as a diagnostic tool in the work-up of women suffering from PND [15–22]. Ductoscopy has been shown to be a useful tool in finding intraductal lesions causing PND (benign and malignant) [23–25]. Next to its diagnostic role, ductoscopy can potentially treat the actual cause of PND as well by mechanical removal [22, 26] or laser ablation [27] of intraductal lesions like papillomas. Therefore, ductoscopy has the ability to replace invasive surgical procedures in patients suffering from PND.

Besides their difference in diagnostic and therapeutic capabilities, major duct excision, MRI and ductoscopy also differ in costs. For example, although effective in the actual treatment of PND, the costs of major duct excision exceed those of MRI and ductoscopy together. So, better selection of patients that actually will benefit from duct excision is crucial to safe costs and to save women from the undesirable side effects of surgery.

As the above shows, there is a need to establish the most cost-effective work-up for women presenting with PND. Therefore, the aim of our study was to compare the diagnostic performance for detecting breast cancer of ductoscopy and MRI in patients with PND in order to better select who is eligible for surgery. Additionally, we performed a cost-effectiveness analysis (CEA) for the diagnostic performance for detecting breast cancer of ductoscopy and MRI, followed by a CEA for the treatment of PND comparing major duct excision and ductoscopy.

## Materials and methods

### Meta-analysis

The systematic literature search on the diagnostic performance of ductoscopy and MRI was performed according to the Preferred Reporting Items for Systematic Reviews and Meta-Analyses (PRISMA) guidelines for meta-analysis [28]. The PubMed and Embase databases were systematically searched for studies published until January 2020. The search strategy was performed on synonyms and medical subject heading (MESH) terms of pathological nipple discharge and the index tests (MRI and ductoscopy). Only articles that evaluated MRI and/or ductoscopy, reported original data and were written in English were selected. Full syntaxes are shown in Supplementary Appendix 1. After removal of duplicates, two authors (MF, SP) independently screened articles by title, abstract and full text. Any disagreement was solved through discussion to reach a consensus.

### Selection of studies

Title/abstract screening was performed after removal of duplicates. Full texts were retrieved for studies that evaluated MRI and/or ductoscopy, reported original data and were written in English.

1. Participants: patients with PND without history of breast cancer or radiological suspicion of breast cancer.
2. Intervention: MRI and/or ductoscopy.
3. Comparator: all patients must have had definitive diagnosis of malignancy by the means of biopsy or histopathological analysis after surgery.
4. Outcome: diagnostic performance of ductoscopy and MRI for the detection of (pre)cancerous lesions.
5. Study characteristics: all studies accepted for publication written in English.

Studies were excluded from systematic review owing to the following reasons:

1. Not possible to determine sensitivity and specificity from the studies by means of true positive, true negative, false positive and true negative.
2. Studies in which none of the patients had histopathological confirmation of malignancy.
3. Case report, review and conference abstracts.

### Risk of bias

The QUADAS-2 Tool was used to evaluate the quality of each eligible study [29]. The entire scale constituted four domains for the risk of bias: patient selection, index test, reference standard and flow and timing. Additionally, there were three domains for applicability concerns: patient selection, index test and reference standard. Each domain could be judged as any of the three levels, low risk, intermediate/unclear risk, or high risk of bias.

Additionally, funnel plots and Egger’s test were performed in order to see whether there was publication or small sampling bias [30].

### Classifications

MRI scans were classified according to the Breast Imaging Reporting and Data System (BI-RADS) reporting system [31]. BI-RADS I–III was considered benign and BI-RADS IV to VI were considered suspicious for malignancy or malignant.

### Cost-effectiveness analysis model

Firstly, a CEA model was developed to capture the costs and effectiveness of ductoscopy, MRI and duct excision surgery. In this model, surgery was performed if ductoscopy or MRI was suspicious for breast cancer. Model outputs were represented in terms of effects of diagnostic success for the detection of breast cancer in patients with PND without suspicion for malignancy on ultrasound or mammography. A random sample of 10,000 patients per diagnostic method was generated with an incidence of 5% based on literature [10, 11]. Analysis was performed with 100 bootstraps.

Secondly, another CEA model was developed to determine the costs and effectiveness of ductoscopy in treating PND. Data were obtained from our previous clinical study [26]. Model outputs were represented in terms of effect of therapeutic success after ductoscopy and/or surgery in patients with PND without suspicion for malignancy on ultrasound or mammography.

A univariate sensitivity analysis was performed using as minimum and maximum values the lower and upper limits of the 95% confidence intervals for the sensitivity of ductoscopy and MRI. In addition, univariate sensitivity analysis was also performed with the different rates of successful ductoscopy according to the literature.

### Cost calculation

Costs of ductoscopy comprised actual staff and equipment costs since ductoscopy is currently not (yet) covered by medical insurance in The Netherlands. The staff costs covered the surgeon performing the ductoscopy, two nurses (one scrub nurse and one circulating nurse) and overhead costs. Equipment costs consisted of ductoscopy materials (hardware and reusables), overhead costs, sterilisation costs of the 0.55 mm optic (LaDuScope T-flex; Polydiagnost) and the Polyshaft 1.15-mm outer diameter, PD-DS-1015; Polydiagnost). The costs were incorporated in a decision model using probabilities of events and unit costs of ductoscopy and MRI [32]. The total costs of surgery were estimated based on average overall hospital costs, including surgical or nonsurgical charges of the UMC Utrecht. All costs are presented in Euros (€), according to the price quotes of 2019.

### Statistical analysis

Firstly, sensitivity and specificity were calculated for ductoscopy and MRI with the 95% confidence interval (CI). After this, pooled estimates of sensitivity and specificity were calculated for ductoscopy and MRI using fixed-effects. Heterogeneity among studies was quantified by the I-square and tested using Cochran’s-Chi-square tests. Subsequently, the chance of a positive test (for MRI and ductoscopy), positive predictive value (PPV) and negative predictive value (NPV) were calculated from the pooled sensitivity, specificity and the prevalence of breast cancer.

Decision trees were modelled using TreeAge Pro V.2015 (TreeAge Software, Williamstown, Massachusetts, USA). All calculations were performed using RStudio 1.2.5001 (with R version: x64 3.6.3). Additionally, statistical packages *meta*, *mada*, *metaphor* and mvmeta were used for all computations and visualisations of the meta-analysis. Cost-effectiveness computations and visualisations were performed using *ICEinfer* package. Finally, other visualisation of plots was done using the *ggplot2* package.

## Results

### Meta-analysis

**Fig. 1:**
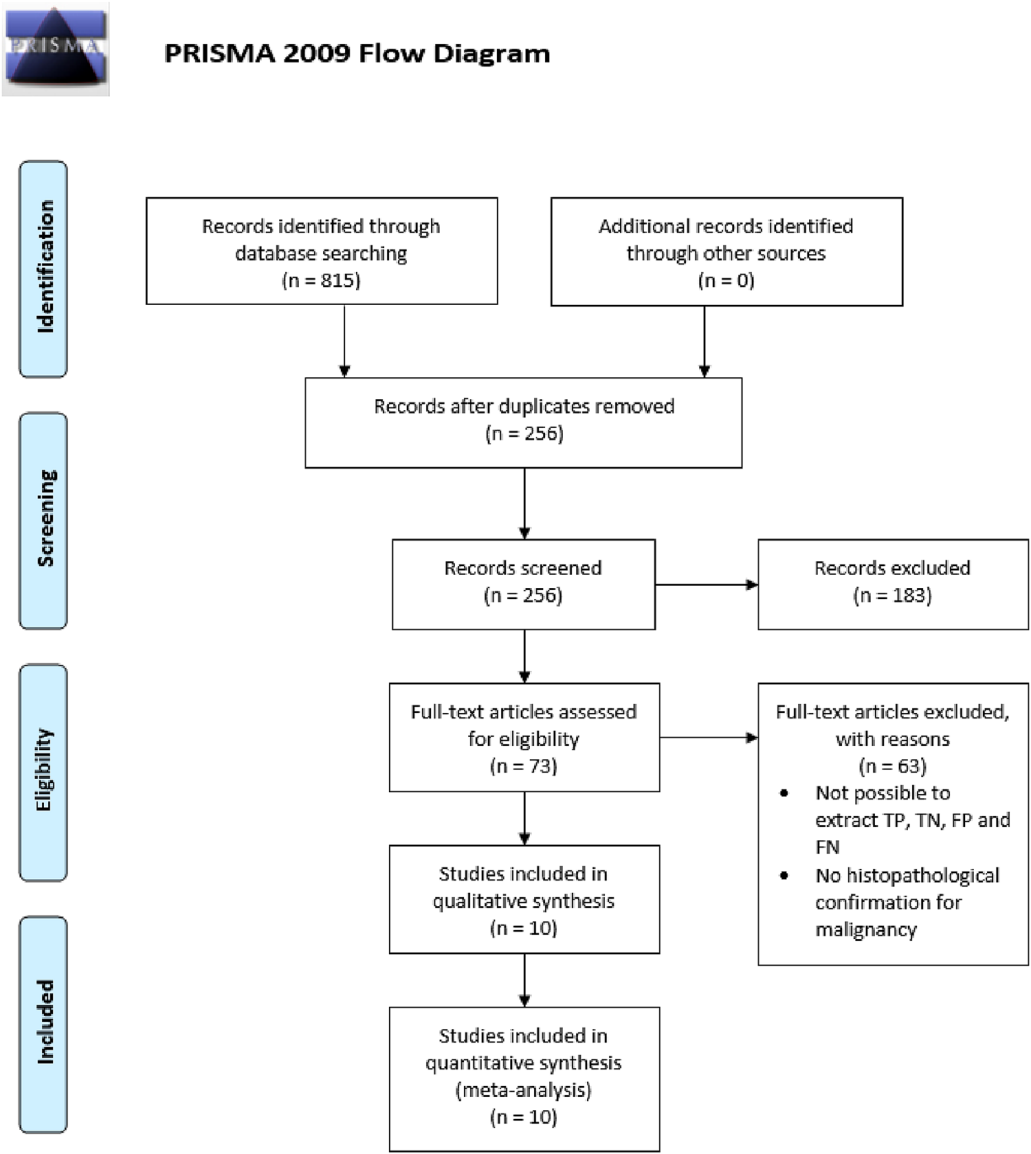
Flow chart showing literature search and study selection with 10 relevant studies ultimately enrolled in the meta-analysis. *N* number, *TP* true positive, *TN* true negative, *FN* false negative, *FP* false positive

A total of 815 citations of articles in English language were identified by the search and, after removing duplicates and screening on relevance, 73 potentially eligible articles were retrieved in full text (Fig. 1). Overall, 894 patients in 10 studies with PND underwent ductoscopy, MRI and/or duct excision surgery. Table 1 shows the details of the studies used in the analysis.

**Table 1:** Baseline characteristics of studies on diagnostic modalities in patients with pathologic nipple discharge without radiological signs of malignancy

Author	Year	Country	Standard method	*N*	Diagnostic modalities
Morrogh et al. [48]	2007	USA	Histopathological diagnosis	33	MRI
Denewer et al. [41]	2008	Egypt	Histopathological diagnosis	53	Ductoscopy
Bender et al. [40]	2009	Turkey	Histopathological diagnosis	102	Ductoscopy
Vaughan et al. [39]	2009	USA	Histopathological diagnosis	89	Ductoscopy
van Gelder et al. [12]	2015	Netherlands	Histopathological diagnosis	107	MRI
Sanders et al. [9]	2016	USA	Histopathological diagnosis	85	MRI
Bahl et al. [49]	2017	USA	Histopathological diagnosis	105	MRI
Gui et al. [42]	2018	UK	Histopathological diagnosis	32	Ductoscopy
Zacharioudakis et al. [33]	2019	UK	Histopathological diagnosis	82	MRI
Filipe et al. [26]	2020	Netherlands	Histopathological diagnosis	206	Ductoscopy

*UK* United Kingdom, *USA* United States of America, *N* total number of patients

**Fig. 2:**
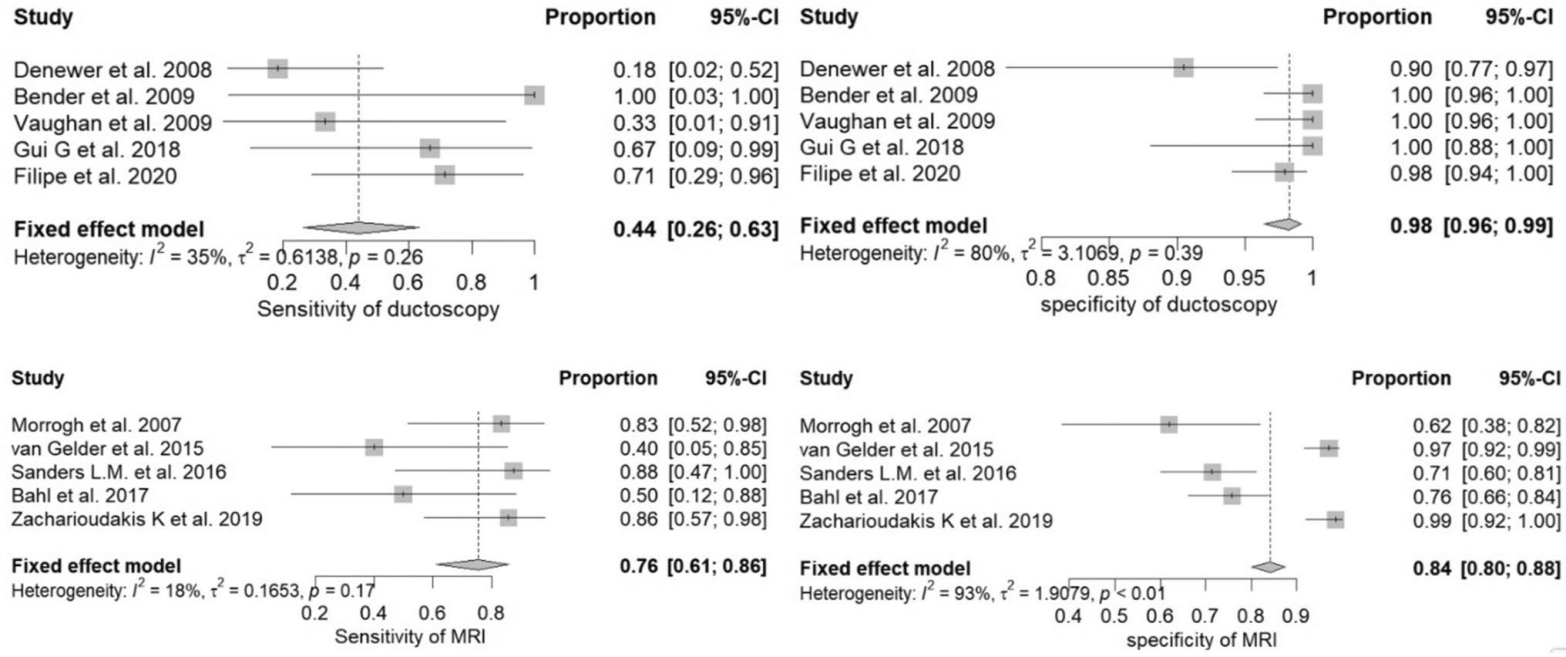
Meta-analysis of sensitivity and specificity of ductoscopy and MRI for detection of breast cancer in patients with pathologic nipple discharge. *MRI* magnetic resonance imaging, *CI* confidence interval

Figure 2 shows the diagnostic performance of ductoscopy and MRI. Ductoscopy had a pooled sensitivity of 44% (95% CI of 22–66%) for detection of breast cancer and a specificity of 98% (95% CI of 96–99%) for the detection of malignancy. Sensitivity and specificity of MRI were 76% (95% CI of 71–86%) and 84% (95% 80–88%), respectively. The prevalence of malignancy in patients with PND without radiological suspicion for malignancy was around 5% [10, 11]. Based on this prevalence, estimated PPV and NPV were 53.7% and 97.1% for ductoscopy, respectively. MRI had an estimated PPV of 20% and a NPV of 98.5%.

The result of the QUADAS-2 tool revealed that all the included studies were of sufficient quality. This was for both risks of bias domains and applicability domains (Supplementary Fig. 1). Additionally, this study showed symmetry of the effect, indicating no evidence for a small sample effect or publication bias in the subgroup analysis (Supplementary Fig. 2). P-values for Egger’s test for sensitivity and specificity were 0.0504 and 0.755, respectively.

### Cost analysis

The input model (Table 2) was based on the data from the meta-analysis of this study, as well as other references and findings of the financial departments of the UMC Utrecht. The average costs of a ductoscopy procedure, a major duct excision operation and a breast MRI at the UMC Utrecht in the year 2019 were €1401.33, €6494.27 and €822.13, respectively.

**Table 2:** Model inputs: clinical and cost parameters (2019, €) of ductoscopy and MRI in patients with PND without radiological signs of malignancy

Parameters	Values	Source
Prevalence of breast cancer	5%	[10, 11]
Ductoscopy parameters		
Sensitivity	44% (95% CI 22–66%)	Study data
Specificity	98% (95% CI 96–99%)	Study data
Successful ductoscopy rate	70.2% (70.2–100%)	Study data
PND stopped after successful ductoscopy	60.3%	[26]
PND stopped after unsuccessful ductoscopy	29.7%	[26]
Costs of ductoscopy	€1401.33	Study data
MRI parameters		
Sensitivity	76% (95% CI 71–86%)	Study data
Specificity	84% (95% CI 80–88%)	Study data
Costs of MRI	€822.13	Study data
Surgery parameters		
PND stopped after surgery	100%	Expert opinion
Costs of surgery	€6494.27	Study data

*PND* pathological nipple discharge, *MRI* magnetic resonance imaging, *CI* confidence interval

### Diagnostic cost-effectiveness analysis comparing ductoscopy to MRI

Based on the diagnostic performance (sensitivity, specificity, PPV and NPV) calculated earlier, Supplementary Fig. 3 shows the CEA of the diagnostic performance of ductoscopy, duct excision surgery and MRI for the detection of cancer in patients with PND with negative conventional radiological findings.

The chance of positive findings at ductoscopy (including unsuccessful ductoscopy procedures) was 4.1%, of which 53.7% were true positive. Consequently, the chance of negative findings at ductoscopy was 95.9% of which 97.1% was true negative. Hence, based on diagnostic performances and costs, the average cost of ductoscopy to diagnose (pre)cancerous lesions, and subsequent surgery when positive, would be €1670.97. The diagnostic accuracy of ductoscopy was 95.3%.

MRI was positive in 19.0% of the cases, of which 20.0% were true positive. Therefore, the chance of a negative MRI was 81.0% of which 98.5% was true negative. Furthermore, the average estimated cost using MRI to diagnose (pre)cancerous lesions, and subsequent surgery when positive, in PND patients would be €2070.27. This based on the fact that 19% of patients with PND without radiological suspicion for malignancy are estimated to have a positive MRI and therefore referred for surgery. This results in a sum of the costs of MRI (€822.13) and the in 19% of the cases surgery (€6494.27). The diagnostic accuracy of MRI was 83.6%. Exact calculations can be found in Supplementary Fig. 3. The current study showed that ductoscopy was more cost-effective for the detection of malignancy in patient with PND compared to MRI, regardless of the margin of error of the sensitivity (95% CI). Sensitivity analysis determining the different cost-effectiveness based on the 95% CI sensitivity of ductoscopy and MRI can be found in Supplementary Table 1.

### Therapeutic cost-effectiveness analysis comparing ductoscopy to surgery

In our previous study 215 patients underwent a ductoscopy procedure. The therapeutic success rate was defined as total relief of PND for at least three months (median follow-up was 14.1 months), regardless of the findings during the ductoscopy itself. The technical success rate of ductoscopy itself was 70.2% (i.e. the procedure could be fully accomplished and sufficient inspection of the ductal tree was possible). A total of 60 patients (27.9%) were operated, for different reasons, in addition to ductoscopy; ductoscopy itself technically failed (*N* = 24), suspicious findings (*N* = 8) or the PND did not stop (*N* = 42). In 60.3% of the technically successful ductoscopy procedures (i.e. inspection of the ductal tree was possible) the PND stopped, of which 7.7% were subsequently operated due to suspicious findings. Consequently, in 39.7% of technically successful ductoscopy procedures the PND did not stop of which 48.3% underwent surgery [26].

Thereby, the effectivity of ductoscopy was 51.2% (percentage of patients that no longer suffered PND) and the average total cost of a patient with PND undergoing a therapeutic strategy with ductoscopy was €3208.89. This cost is based on the sum of the cost for ductoscopy (€1401.33) and the fact that 48.3% of patients that underwent ductoscopy also underwent subsequent duct excision surgery (€6494.27). Exact calculations can be found in the Supplementary Fig. 4. The current study showed that ductoscopy was more cost-effective for the treatment of PND compared to duct excision surgery, regardless cannulation rates reported by the literature. Sensitivity analysis determining the different cost-effectiveness based on the cannulation of ductoscopy according to the literature can be found in Supplementary Table 2.

## Discussion

In this study, a meta-analysis was performed on the diagnostic performances of ductoscopy and MRI for the detection of (pre)cancerous lesion in patients with PND without radiological suspicion for malignancy. This study also shows the results of a CEA comparing ductoscopy to MRI in this group of patients for the detection of malignancy and also their capability to select patients for major duct excision. Finally, we performed a CEA comparing ductoscopy to major duct excision for the therapeutic effect in resolving PND in these patients.

For the meta-analysis, 10 studies were finally selected, which together included a total of 894 patients suffering from PND. We compared ductoscopy to MRI in terms of sensitivity and specificity. Pooled sensitivity and specificity of ductoscopy were 44% and 98%, respectively. MRI showed a pooled sensitivity of 76% and 84%, respectively. There were no statistically significant differences in sensitivity, but specificity was statistically significantly higher for ductoscopy.

In recent years, MRI has been used more often for the detection of breast cancer in patients with PND. Based on our meta-analysis, MRI has a high sensitivity for the detection of breast cancer in this group. However, due to a relatively low specificity, histopathological assessment through surgery or biopsy remains necessary to determine whether the lesion is benign-or-not [13, 14]. MRI is also increasingly used in cases of PND when mammography and ultrasound are negative [8, 12, 33, 34]. The current study clarifies that MRI has a higher sensitivity (although not significant) but shows at the same time a statistically significantly lower specificity in comparison to ductoscopy for the detection of breast cancer in patients with PND. Contrast enhanced MRI appears to be a promising imaging method for the detection of breast cancer in this group of patients [35–37]. However, contrast enhanced MRI studies in PND patients are scarce and include only few patients for which reason they were not included in our meta-analysis [35–37].

Over the last few decades, ductoscopy has been gaining ground for detection of lesions causing PND [38–43]. The meta-analysis performed in this study shows that ductoscopy has a similar (not significant) sensitivity (44 vs 86%) but a significantly higher (84 vs. 98%) specificity in comparison with MRI. However, since the prevalence of (pre)cancerous lesions is only around 5% in patients suffering PND without radiological signs of malignancy, specificity is a more useful tool to determine diagnostic performance. This is also reflected by the fact that our study shows that the diagnostic accuracy of ductoscopy (95.3%) is significantly higher compared with MRI (83.6%). Therefore, it is safe to conclude that ductoscopy seems to be a more useful tool to determine which patients are eligible for (duct excision) surgery. This is in line with previous studies that showed that ductoscopy successfully reduces the need for surgery in patients with PND [26, 44]. Additionally, since intraductal extractions are nowadays possible with the basket extraction device and pilot studies with laser ablation have been done [26, 45], ductoscopy also has a therapeutic potential in the treatment of PND itself [22], making it an even more attractive modality.

In our study we also show that ductoscopy is more beneficial (11.9%) and less costly (€399.30) compared with MRI for the proper selection of PND patients for surgery. This can be explained by the specificity of MRI and ductoscopy. With a malignancy prevalence of 5%, the significantly higher specificity of ductoscopy ensures that only 4.1% of ductoscopy procedures are positive (regardless whether it is true positive of false positive). This means that only 4.1% of patients will undergo surgery when ductoscopy is used. However, since specificity of MRI is significantly lower (84%), despite the fact that sensitivity is higher, the chance of a positive MRI is 19%. This means that 19% of patients with PND without radiological suspicion will undergo surgery. Consequently, when a MRI is performed, chances of a PND patient undergoing surgery is almost 5 times higher. Therefore, even though a single MRI is less costly than a ductoscopy (€822.13 vs. €1401.33, respectively), this analysis shows that it would still be considerably less expensive to use ductoscopy as a strategy in determining the need for surgery in this patient population. Multivariate sensitivity analysis taking the uncertainties of the sensitivities for the detection of malignancy in patients with PND with negative conventional imaging of ductoscopy and MRI into account showed no significant changes to these conclusions.

As mentioned above, ductoscopy (unlike MRI) also has a potential therapeutic effect on the PND itself. This results in a further decrease in the number of major duct excisions needed. Based on the clinical data from our previous published study we showed that in over half (51.7%) of patients undergoing (attempted) ductoscopy the PND actually stopped and only 27.9% of women suffering PND finally needs surgery after ductoscopy [26].

Our study also has some limitations. First, the diagnostic section was modelled, based on pooled diagnostic performances of ductoscopy and MRI. However, there was an unexplained difference in the prevalence of malignancy between ductoscopy (4.5%) and MRI (19%) studies. This might explain the high heterogeneity for specificity in both ductoscopy and MRI. Since there is a consensus that the malignancy rate in patients with PND without radiological signs of malignancy is around 5% [14, 24], the current study parted from that premise and did not include prevalence in the sensitivity analysis. Second, in the included studies there are different definitions of a technically successful ductoscopy. This study defined successful ductoscopy as being able to visualise the ductal tree. However, other studies defined successful ductoscopy as being able to cannulate the ductal tree, regardless of being able to visualise-or-not. We performed a sensitivity analysis for this uncertainty but this did not change the conclusions. Thirdly, since most intraductal lesions causing PND are directly behind the nipple, biopsy is often not possible and surgery is recommended [46, 47]. For this reason, costs of biopsy were not taken into account in this study. Fourthly, all costs were obtained from only one hospital, since the UMC Utrecht is the only hospital in The Netherlands performing ductoscopy. Although costs for medical procedures might differ from one hospital to another, it does not seem very likely that the ratio between these costs within one hospital will vary much. Therefore, the effect on our analysis is probably limited. At last, there are currently no studies describing the quality of life of patients with PND undergoing MRI, ductoscopy or duct excision surgery. For that reason, quality of adjusted life analysis was not used in this study.

To conclude, this study is the first to directly compare the diagnostic performance of ductoscopy and MRI in patients with PND without radiological signs of malignancy. This study shows that ductoscopy has a significantly higher diagnostic accuracy in this patient population. This makes ductoscopy less costly and a more effective diagnostic tool in comparison with MRI to determine which patients finally require surgery to rule out malignancy. Furthermore, this CEA showed that, while ductoscopy is not as effective in treating PND as duct excision surgery, it is much less costly.

## Electronic supplementary material

Below is the link to the electronic supplementary material.

Electronic supplementary material 1 (DOCX 688 kb)
