# Supplementary material for: Meta-analysis and cost-effectiveness of ductoscopy, duct excision surgery and MRI for the diagnosis and treatment of patients with pathological nipple discharge
Source: Breast Cancer Res Treat. 2021 Jan 21;186(2):285–93. doi: 10.1007/s10549-021-06094-x (PMC7990840; doi:10.1007/s10549-021-06094-x)
Supplement: Supplementary file 1 — Electronic supplementary material 1 (DOCX 688 kb) [file 10549_2021_6094_MOESM1_ESM.docx]

Title:

Cost-effectiveness analysis, systematic review and meta-analysis of ductoscopy, duct excision surgery and MRI for the diagnosis and treatment of patients with pathological nipple discharge

Authors

M.D. Filipe^1^ MD [m.d.filipe-2@umcutrecht.nl](mailto:m.d.filipe-2@umcutrecht.nl)

E. Postma ^2^ MD PhD

T. van Dalen^3,4^

M.R. Vriens^1^ MD PhD

P. van Diest^2^ MD PhD

A.J. Witkamp^1^ MD PhD [a.j.witkamp@umcutrecht.nl](mailto:a.j.witkamp@umcutrecht.nl)

^1^ Department of Surgical Oncology, Cancer Centre, University Medical Centre, the Netherlands

^2^ Departments of Pathology and Oncology, University Medical Centre, the Netherlands

^3^ Cochrane Netherlands, University Medical Center Utrecht, Utrecht, the Netherlands

^4^Julius Center for Health Sciences and Primary Care, University Medical Center Utrecht, Utrecht University, the Netherlands

Corresponding author

M.D. Filipe

Address: PO Box 85500, 3508 GA, Utrecht, the Netherlands

E-mail: [m.d.filipe-2@umcutrecht.nl](mailto:m.d.filipe-2@umcutrecht.nl)

# Appendix 1: Search strategy

**Pubmed**

((("Nipple Discharge"[Mesh]) OR nipple discharge*[Title/Abstract])) AND (((OR "Mammography"[Mesh] OR "Magnetic Resonance Imaging"[Mesh] OR "Endoscopy"[Mesh] OR "ductoscopy"[tiab] OR "ductoscope"[tiab])) OR ((Magnetic Resonance Imaging[Title/Abstract] OR MRI[Title/Abstract] OR endoscop*[Title/Abstract] OR ductoscop*[Title/Abstract] OR fiberoductoscop*[Title/Abstract] OR FDS[tiab])))

**Embase**

('breast discharge'/exp OR 'breast discharge*':ti,ab,kw) AND ('nuclear magnetic resonance imaging'/exp OR 'nuclear magnetic resonance imaging':ti,ab,kw OR 'ductoscop*':ti,ab,kw OR 'endoscopy'/exp OR 'endoscopy':ti,ab,kw OR 'fiberoductoscop*':ti,ab,kw)

# Appendix 2: Supplementary Figures

| 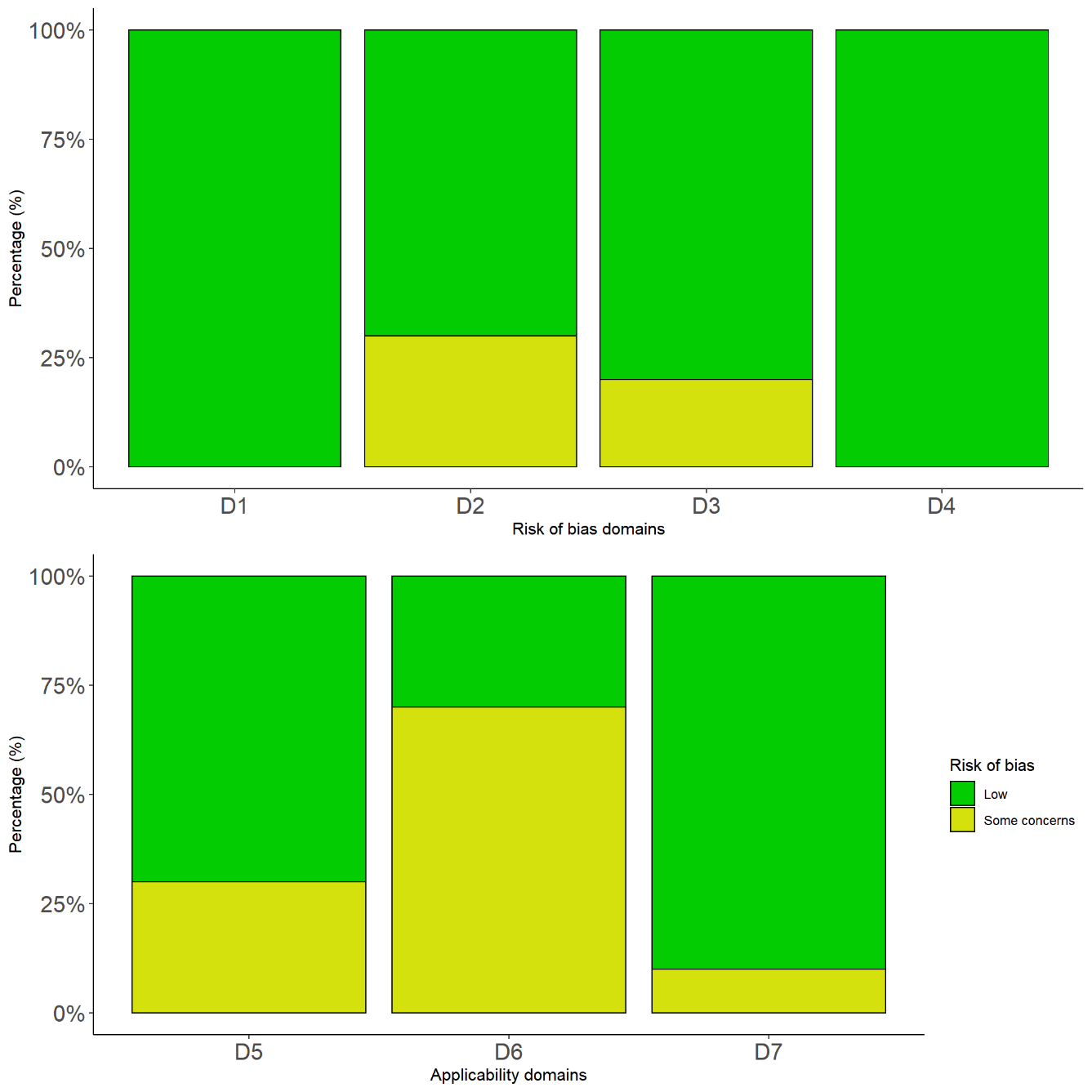 |
| --- |
| **Supplementary figure 1.** Summary of the risks of bias and applicability domains. D1= Patient selection; D2 = Index test; D3 = Reference standard; D4 = Flow and timing; D5 = Patient selection; D6 = Index test; D7 = Reference standard. |

| 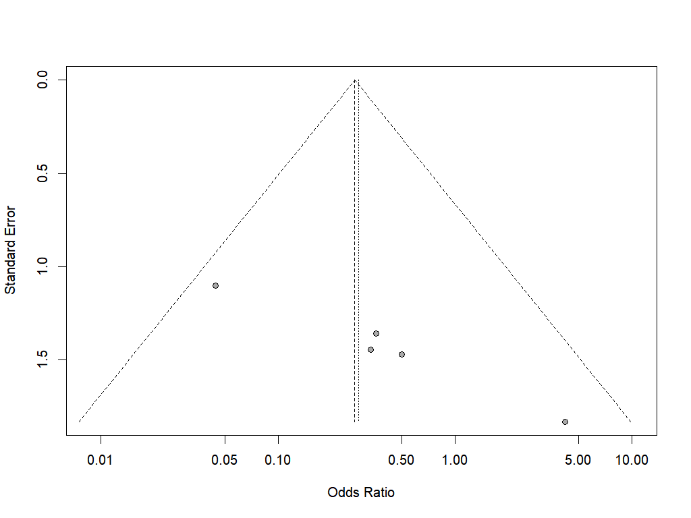 |
| --- |
| 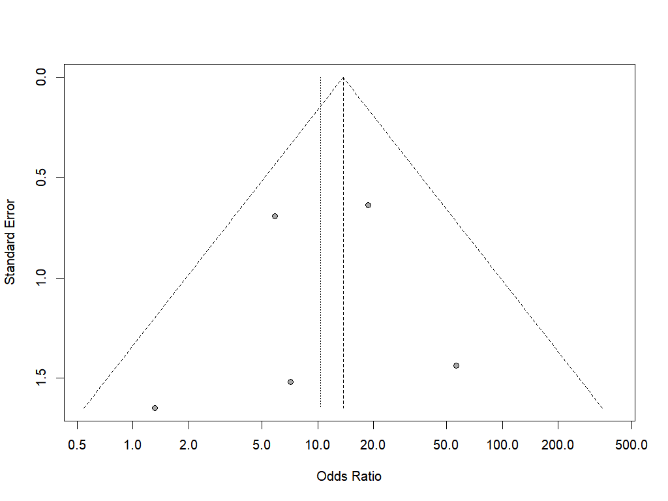 |
| **Supplementary figure 2.** Funnel plots for sensitivity (top) and specificity (bottom) comparing MRI to ductoscopy in patients with PND without radiological suspicion for malignancy. MRI = Magnetic resonance imaging, PND = Pathological nipple discharge. |

| \| 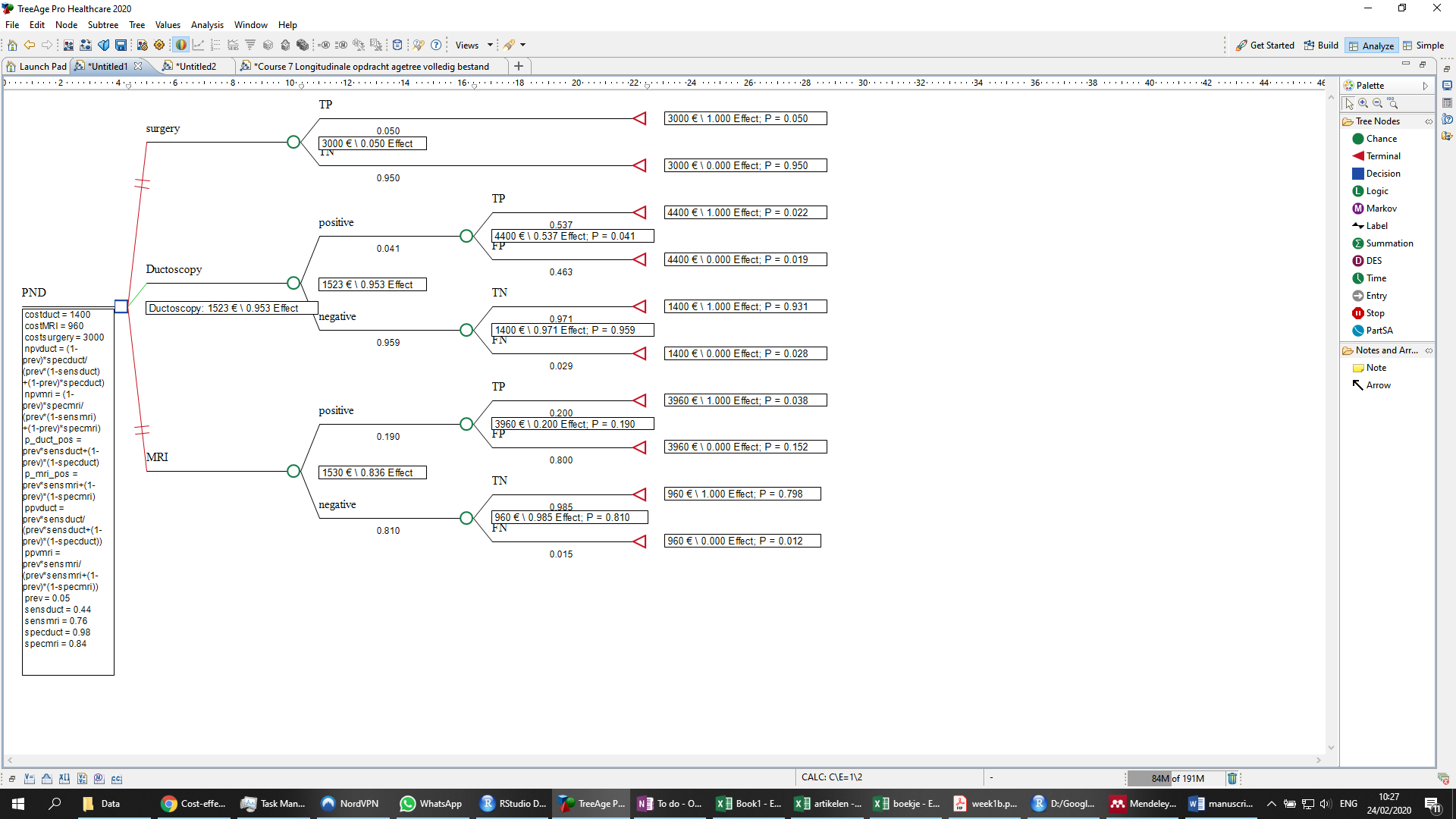 \| \| --- \| \| **Supplementary Figure 3.** Cost-effective analysis of diagnostic performance of ductoscopy, duct excision surgery and MRI for the detection of cancer in patients with PND with negative conventional radiological findings. PND = Pathological nipple discharge, MRI = magnetic resonance imaging, TP = true positive, FP = False positive, FN = False negative, TN = True negative. \| |
| --- | --- | --- |
|  |

| 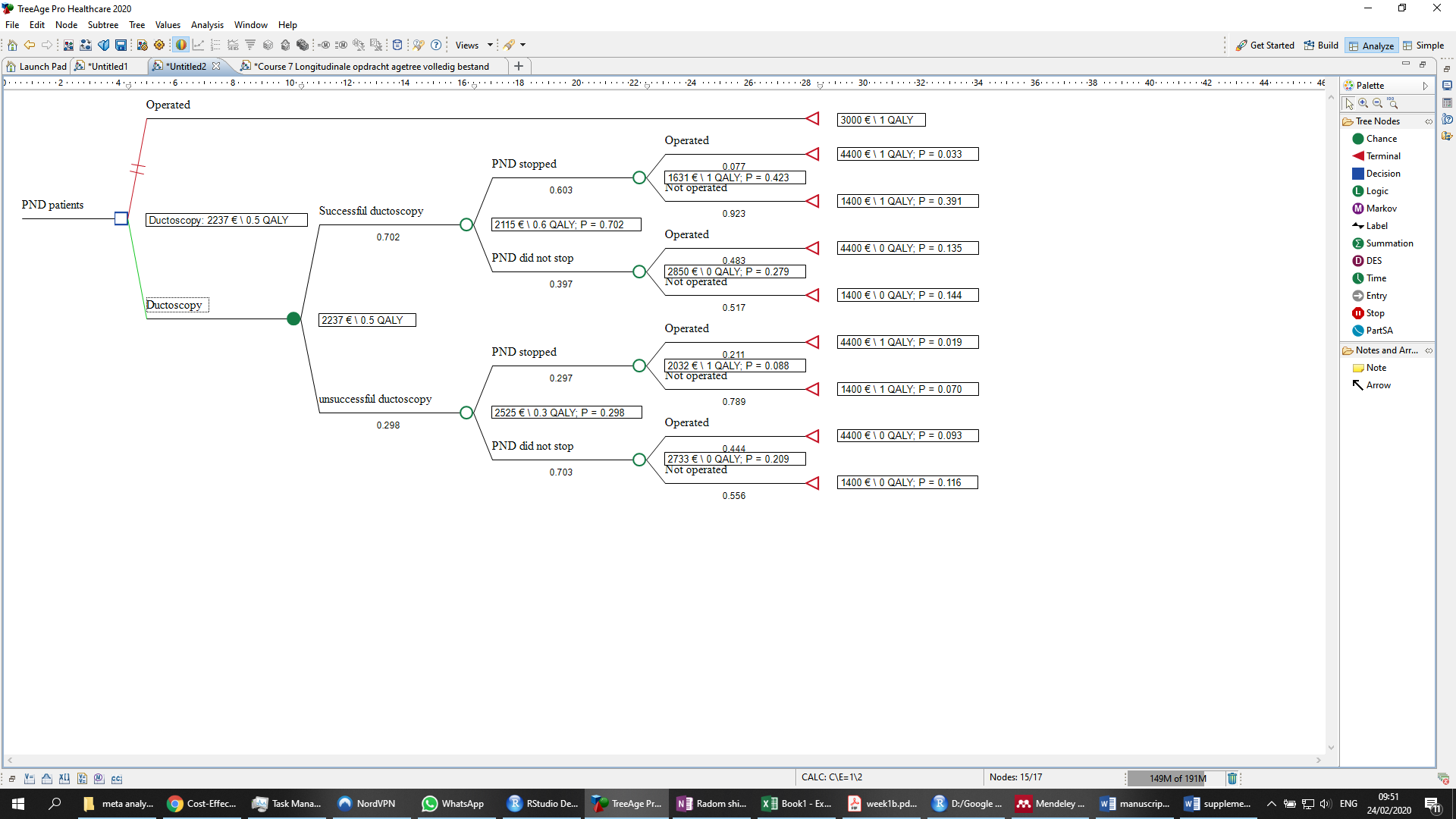 |
| --- |
| **Supplementary Figure 4.** Cost-effective analysis of therapeutic performance of ductoscopy and duct excision surgery in patients with PND with negative conventional radiological findings. PND = Pathological nipple discharge |

# Supplementary tables

| **Supplementary Table 1.** Different ICER depending on the sensitivities (based on the 95% CI) of ductoscopy and MRI for the detection of breast cancer in patients with PND without radiological signs of malignancy. | | | | |
| --- | --- | --- | --- | --- |
| **Sensitivity of Ductoscopy/MRI** | **71%** | **76%** | **81%** | **86%** |
| 21% | -4781.8 | -5061.65 | -5301.66 | -5636.13 |
| 33% | -4305.5 | -4551.07 | -4773.68 | -5082.32 |
| 44% | -3815.41 | **-4026.45** | -4230.66 | -4512.16 |
| 55% | -3375.32 | -3557.04 | -3745.16 | -4003.06 |
| 66% | -2961.47 | -3116.97 | -3290.14 | -3526.26 |
| MRI = Magnetic resonance imaging, ICER = Incremental cost-effectiveness ratio, PND pathological nipple discharge, CI = confidence interval. Bold is the ICER when pooled sensitivity of ductoscopy and MRI are used. | | | | |

| **Supplementary Table 2.** Cost-effectiveness analysis comparing ductoscopy to duct excision surgery in patients with PND without radiological signs of malignancy based on the different reported cannulation rates in the known literature. | | | |
| --- | --- | --- | --- |
| **Ductoscopy success rate** | **Effect difference** | **Cost difference** | **ICER** |
| 70.2% | -0.39703 | -3441.13 | 8680.677 |
| 75% | -0.40015 | -3454.84 | 8649.111 |
| 80% | -0.39941 | -3462.60 | 8680.545 |
| 90% | -0.39920 | -3509.62 | 8800.454 |
| 95% | -0.39590 | -3544.97 | 8968.017 |
| 100% | -0.40092 | -3536.62 | 8831.185 |
| ICER = Incremental cost-effectiveness ratio, PND pathological nipple discharge. | | | |
